# Health information seeking on the Internet: a double divide? Results from a representative survey in the Paris metropolitan area, France, 2005–2006

**DOI:** 10.1186/1471-2458-8-69

**Published:** 2008-02-21

**Authors:** Emilie Renahy, Isabelle Parizot, Pierre Chauvin

**Affiliations:** 1 INSERM, U707, Research Team on the Social Determinants of Health and Healthcare, Paris, F-75012, France; 2 Université Pierre et Marie Curie-Paris6, UMR-S 707, Paris, F-75012, France; 3 AP-HP, Hôpital Saint Antoine, Department of Public Health, Paris, F-75012, France

## Abstract

**Background:**

The Internet is a major source of information for professionals and the general public, especially in the field of health. However, despite ever-increasing connection rates, a digital divide persists in the industrialised countries. The objective of this study was to assess the determinants involved in: 1) having or not having Internet access; and 2) using or not using the Internet to obtain health information.

**Methods:**

A cross-sectional survey of a representative random sample was conducted in the Paris metropolitan area, France, in the fall of 2005 (n = 3023).

**Results:**

Close to 70% of the adult population had Internet access, and 49% of Internet users had previously searched for medical information. Economic and social disparities observed in online health information seeking are reinforced by the economic and social disparities in Internet access, hence a double divide. While individuals who reported having a recent health problem were less likely to have Internet access (odds ratio (OR): 0.72, 95% confidence interval (CI): 0.53–0.98), it is they who, when they have Internet access, are the most likely to search for health information (OR = 1.44, 95% CI = 1.11–1.87).

**Conclusion:**

In the French context of universal health insurance, access to the Internet varies according to social and socioeconomic status and health status, and its use for health information seeking varies also with health beliefs, but not to health insurance coverage or health-care utilisation. Certain economic and social inequalities seem to impact cumulatively on Internet access and on the use of the Internet for health information seeking. It is not obvious that the Internet is a special information tool for primary prevention in people who are the furthest removed from health concerns. However, the Internet appears to be a useful complement for secondary prevention, especially for better understanding health problems or enhancing therapeutic compliance.

## Background

The Internet access rate has continued to increase in all industrialised countries over the past 15 years, having climbed recently to 54% in France and 70% in the United States [1]. The Internet is now a major information tool, both in people's professional and private lives. However, several studies report the existence of a persistent digital divide both in Internet access [2-4] and Internet use [5]. Such is the context in which a large number of general public health-related websites and discussion groups have been created. Many studies have been carried out to describe the characteristics of people who use the Internet to obtain health information. Most of them indicate that the discriminating factors are age, level of education, level of income and ethnicity [6]. Online health seekers are more likely to be young, to have a higher level of education, a higher level of income, and to be white (in the United States). The same differences have been shown for Internet access as such [2-4]. Other studies have also suggested an association with long-term illness [7], reported chronic conditions [8] or a greater health concern [9]. Moreover, some researchers have suggested that online health information especially benefits the already privileged in terms of health, health-care utilisation or socioeconomic status [10-12]. Some patient population-based studies, have led to a better understanding of the circumstances of online health information seeking in terms of the stage of an illness [13,14]. Other studies also debate the actual benefits of the Internet in general and online discussion groups in terms of the social support (positive most of the time [15,16], but there is no robust evidence [17]).

Most of these studies have involved patients with specific diseases (e.g., cancer or HIV infection) or small and/or nonrandom samples. Moreover, many of them are purely descriptive or examine only one explanatory dimension, and none of them have been conducted in France [6]. The probable cumulative impact of barriers to the Internet access and, consequently, to the use of the Internet for health purposes has not, as such, often been studied. Empirical studies are starting to take into consideration more broadly the cognitive barriers to the general public's use of the Internet for health information seeking, including the different skills required to read, use new technologies, search for information, and understand health information (e-health literacy) [18,19]. It seemed important to us to statistically analyze in the same representative population-based survey (and especially in the French context of universal health insurance) the factors associated with Internet access and the use of the Internet to search for health information, both potentially involving common skills and determinants.

The objectives of this study were to describe and compare the determinants involved in: 1) having or not having Internet access; and 2) using or not using the Internet to obtain health information (within the Internet user population). To answer these questions as accurately as possible, several hypotheses were proposed. Apart from the usual characteristics for evaluating the association with demographics and socioeconomic status, we simultaneously tested the potential effect of health status, health perceptions and behaviours, health-care system utilisation, social integration, psychological capital, living conditions, and lastly, technological skills.

## Methods

### Study sample

The SIRS (French acronym for health, inequalities and social ruptures) survey was conducted in the fall of 2005 in a representative sample of the adult French-speaking population in the Paris metropolitan area (Paris and its suburbs, a region with a population of 6.5 million). This survey constituted the first phase of a socio-epidemiological cohort study in the general population, a collaborative research project between the French National Institute for Health and Medical Research, National Centre for Scientific Research and National Institute for Demographic Studies. The sampling was carried out in three stages. We used a geographical partition of the Paris metropolitan area, specifically, the smallest unit area available in the French statistical data system (each neighbourhood has approximately 2000 inhabitants). We first used the Surveyselect procedure (SAS^® ^V9.1) to randomly select 50 neighbourhoods (overrepresenting the poorer neighbourhoods [20]). We then randomly selected households in each neighbourhood. Lastly, we selected the interviewees by the birthday method. No incentives were offered. On the contrary, the survey was clearly presented as a voluntary one conducted by a public research institute. The interviewers were required to visit each household eight times at different dates and times before scratching it off and replacing it with another (chosen at random). Twenty-one percent of the "original" households had to be replaced after eight visits because there was no one at those addresses. In all, 29% of the people contacted declined to answer the survey and 5% were excluded because they did not speak French (3%) or because they were too sick to answer our questions (2%). A questionnaire was administered in person during home visits to a random sample of 3023 people. Data on numerous social and health-related characteristics were collected, as well as answers to a specific module of questions on Internet use.

### Variables of interest

Two outcomes were defined for this study. For Internet use, we considered the fact of personally having gone online, regardless of the location (Yes/No). We subsequently identified among the respondents who had gone onto the Internet those who had used it at least once in the previous three years (Yes/No) to search for information on any of the following health-related topics: medical news, treatment centres, hospitals or physicians, a particular disease or treatment, alternative medicines, or health-related administrative measures. In this paper, health is therefore to be understood in its 'medical' sense (the concepts of nutrition or, more generally, well-being were not listed among the topics selected for our purposes here), while online sources were considered globally, without making any distinction between professional or general public sites.

### Explanatory variables

In addition to age and gender, we distinguished between French people born of two French parents, those born of at least one foreign parent, and foreigners in order to take a certain amount of cultural diversity into account. The respondents' socioeconomic status was characterised by the following variables: level of monthly household income (range: 60–10,000 € per consumption unit; quartile values: 1055/1600/2380), level of education, occupational status and health insurance status. The monthly household income was calculated with the OECD standard method to assess the financial status of people, taking account of the size and composition of households. We added up the individual incomes of all the members of the household. We then divided this sum by an adjusted number of people living in the household or "consumption unit". This number was as follows: the reference person in the household (the one with the highest SES) was assigned a value of 1, every other person over 14 years of age a value of 0.5, and children under 14 a value of 0.3 [21]. With regards occupational status, we differentiated between those who were active (in France, a person is considered active if he or she is working or looking for work), those who were inactive (retired or with no profession), and students. One question on the perception of financial difficulties and another on difficulty reading French rounded out this assessment of socioeconomic status.

Social integration was measured in terms of both family and social integration: civil status (lives or does not live in a couple relationship), number of children, and status in the support network. An individual was considered a "helper" if he or she had helped someone financially or by providing moral support or assistance in daily living during the previous six months; "helped" if he or she had received such help without giving any in return; or "isolated" from the support network if he or she had not given or received any help in the previous six months.

To discern disease experience, we took into consideration the respondents' health status (recent and past) and that of their close relatives. An individual was considered to have been sick in the recent past if he or she reported having had at least one chronic problem in the previous 12 months (based on a preestablished list of the main chronic problems commonly used in other health surveys in France, such as asthma, diabetes, hypertension, cancer, etc. [22]) and/or as being treated or followed on a regular basis for medical reasons and/or as having a disability or handicap. A past disease experience was defined as a report of a chronic problem (on the same list) prior to the previous 12 months and/or a report of "serious health problems" before the age of 18 years. As for the health status of close relatives, we distinguished between a past experience (an illness in the spouse and/or a serious health problem in the parents before the age of 18 years) and a present experience (an illness or a handicap in a member of the household and/or in a close relative on the day of the survey). This different approach for the time frame for measuring health among self and others is due to using secondary data and based upon the available questions.

Additionally, declarative data on different characteristics were collected in order to understand the potential effect of health-care system utilisation: having a regular physician, having been or a close relative having been the victim of a medical error (during lifetime), having used traditional medicine, or having forgone medical care for financial reasons during the previous 12 months.

The respondents' psychological status was evaluated on the basis of the following elements: the presence of an anxiodepressive syndrome on the day of the survey (score determined by the MINI-Diag questionnaire [23], a 10-item questionnaire exploring the presence of problems such as sadness, fatigue, a depressed feeling, a loss of appetite, sleeping disorders, a loss of self-confidence, and so on during the last two weeks, and then dichotomised in a binary depression variable – Cronbach's alpha value of 0.60 for the 10-items and 0.73 for the 3 first ones used as conditional response); perceived mental health on the day of the survey ("In general, would you say that your psychological and emotional health is very good, good, somewhat good, fair or poor?"); and self-esteem (score on the Rosenberg Self-Esteem Scale [24] – Cronbach's alpha = 0.83 – expressed in terms of a quartile).

The respondents were asked about their health perceptions [25] and beliefs about medicine and fate. With regard to perceptions, they were asked about three dimensions: resistance to illness ("My body seems to resist illness very well."), health outlook ("In the future, I expect to have better health than other people I know."), and health worry ("I worry about my health more than other people worry about theirs."). As for beliefs, two questions were asked: "Do you think that medicine has effective solutions to all health problems?" and "Do you think that disease and healing depend on God, fate or providence?". Lastly, we considered whether or not they thought that "information provided by physicians is difficult to understand" or that "health advice and recommendations are difficult to apply in daily life". All the variables were dichotomised between "Yes" (if the respondent was strongly or somewhat in agreement with the statement) and "No" (if he or she was strongly or somewhat in disagreement).

Lastly, the Internet users were asked whether they had a home connection, how often they used the Internet (low versus high, low being defined as every month or less often, high as every day or week) and for how long they had been using it (in years).

### Statistical methods

Since we did not have an *a priori *hypothesis, we decided to estimate the models that fit our data and explain the outcomes the best. The variables potentially associated with the different outcomes were selected one by one, on the basis of "univariate" logistic regression models systematically adjusted for gender, age, level of education and level of income. All the variables significant at a threshold of 0.20 (Wald test) were entered into a multivariate model. We then selected the best model by a manual step-by-step backward procedure (threshold of significance set at 0.05) [26]. We tested all the plausible interaction (but none were found to be significant). We also calculated the Cox and Snell generalized coefficient of determination and the Nagelkerke adjusted coefficient of determination (reaching a maximum of 1) to assess the proportion of variance explained by the final model [27] and the area under the receiver operating characteristic (ROC) curve to provide a more easily interpretable criterion of our model assessment.

All of these statistical analyses were weighted in order to take into account the sampling technique (3-stage sample: census tracts, households and individuals, with initial overrepresentation of the disadvantaged areas) and the poststratification adjustment for age and gender according to the general population census data [28]. The Surveylogistic procedure (SAS^® ^V9.1) was used to take this weighting into account when estimating the parameters, confidence intervals and statistical tests. Lastly, to assess the stability of our results, an automated logistic regression and variable selection procedure was carried out on 200 bootstrap samples generated from our initial sample [29].

## Results

The participation rate in the survey was 71%. The sample consisted of 1423 men (47.1%) and 1600 women (52.9%). With regard to socioeconomic status, 65.9% were active, 27.2% were inactive and 6.9% were students. The median age was 42 years (interquartile range: 30–58). The distribution of the weighted sample by gender, age and socio-occupational status was representative of the adult population in the Paris metropolitan area.

### Internet access

Close to 70% of the respondents had previously personally gone online. We did not consider for this analysis the covariates that pertain to the following dimension: health-care utilisation, health perceptions and Internet use. Although men (73%) seemed to have greater access to the Internet than women (67%), the difference was not significant (Table 1). On the other hand, Internet access decreased significantly with increasing age and increased with the level of education and income. These disparities were reinforced by the perception of financial difficulties and difficulties reading French. When adjusted for all the variables in the model, the French respondents with French parents had a greater likelihood of previously having gone online than those with foreign parents or than foreigners. Individuals with inactive occupational status had less access to the Internet than those with active status, while students had greater access. In addition, the probability of having used the Internet was significantly greater in individuals who were healthy and well integrated into the support network. The generalized and adjusted coefficients of determination in the final model (respectively, R^2 ^= 0.43 and ${\tilde{\text{R}}}^{2}$ = 0.61) and the area under the ROC curve (c = 0.907) showed that the model had very-good-quality prediction and discrimination. In the 200 bootstrap samples, the 10 variables in the model presented here (the four adjustment variables were not taken into account) were the 10 most frequently selected variables (including seven with a frequency greater than 70%).

**Table 1:** Internet access: multivariate logistic regression (Number of observations = 3023)

	**% of sample**	aOR*	**95% CI**	P**
**Gender**				0.761
Men	47.1	1	-	
Women	52.9	0.96	0.74–1.25	
**Age group (years)**				< 0.0001
> 59	22.5	1	-	
45–59	23.7	4.49	2.83–7.12	
30–44	30.4	11.72	7.16–19.19	
< 30	23.4	23.99	13.33–43.16	
**Level of education**				< 0.0001
None/Primary	9.7	1	-	
Secondary	38.9	3.31	2.08–5.29	
Postsecondary	51.4	14.99	9.10–24.71	
**Monthly household income (€/CU)**				< 0.0001
1^st ^quartile	25.0	1	-	
2^nd ^quartile	25.9	1.55	1.12–2.16	
3^rd ^quartile	24.2	2.78	1.82–4.25	
4^th ^quartile	24.9	3.45	2.14–5.58	
**Occupational status**				< 0.0001
Active	65.9	1	-	
Inactive	27.2	0.41	0.27–0.61	
Student	6.9	3.57	1.02–12.63	
**Nationality**				0.002
French/French parents	68.4	1	-	
French/Foreign parents	17.7	0.79	0.56–1.13	
Foreign	13.9	0.49	0.33–0.72	
**Perception of having financial difficulties**				0.008
No	55.0	1	-	
Yes	45.0	0.68	0.51–0.90	
**Difficulty reading French**				< 0.0001
No	91.8	1	-	
Yes	8.2	0.26	0.16–0.43	
**Social integration**				< 0.0001
Isolated	16.7	1	-	
Helped	6.2	1.33	0.73–2.43	
Integrated	77.1	2.60	1.89–3.59	
**Current health problem(s)**				0.036
No	30.3	1	-	
Yes	69.7	0.72	0.53–0.98	

* Adjusted odds ratio

** Overall p_value based on the Type III Wald statistic

### Use of the Internet to search for health information

The most common source of health information mentioned by the survey's respondents was their physician (77% consulted a physician "quite often" or "in most cases" when they had a health question), while 34.2% of the total – or 48.5% of the Internet users – indicated that they had searched for medical information online during the previous three years.

The final multivariate model did not show, among Internet users, an association between health information seeking and occupational status, nationality, financial difficulties or difficulties reading French. We still observed significant associations with age, level of income, level of education, and social integration (Table 2). Actually, the level of education is globally significant in our study (even though none of the education levels were found to be significant). When this variable is dichotomised (postsecondary education versus the other two levels combined), the effect is indeed highly significant (odds ratio (OR) = 1.65, 95% confidence interval (CI) = 1.25–2.17), and estimates of other parameters do not differ. The final model also showed that women were significantly more likely to have previously searched for health information than men (OR = 1.64, 95% CI = 1.29–2.07). In addition, Internet users who were sick or who had a poor perception of their mental health were more likely to have previously searched for health information online (respectively, OR = 1.44, 95% CI = 1.11–1.87, and OR = 1.41, 95% CI = 1.05–1.91). The same was true for individuals who had a close relative with a health problem at the time of the survey. More generally, worrying about one's health more than most people or having difficulty understanding information provided by physicians was positively associated with online health information seeking. Lastly, experience with the Internet (regular use, home connection, and number of years with Internet access) significantly increased this specific use. With generalized and adjusted coefficients of determination of 0.20 (R^2^) and 0.25 (${\tilde{\text{R}}}^{2}$), respectively, and an area under the ROC curve of 0.73, the model's prediction quality was acceptable. In addition, the variables are those found most often in bootstrap modelling (eight variables in more than 65% of the cases).

**Table 2:** Use of the Internet for health information seeking: multivariate logistic regression (Number of observations = 1931)

	**% of Internet users**	aOR*	**95% CI**	P**
**Gender**				< 0.0001
Men	49.0	1	-	
Women	51.0	1.64	1.29–2.07	
**Age group (years)**				< 0.001
> 59	10.6	1	-	
45–59	22.8	1.54	1.01–2.36	
30–44	36.2	1.81	1.19–2.75	
< 30	30.4	2.73	1.71–4.38	
**Level of education**				0.001
None/Primary	1.4	1	-	
Secondary	32.5	1.47	0.60–3.60	
Postsecondary	66.1	2.39	0.97–5.85	
**Monthly household income (€/CU)**				0.020
1^st ^quartile	25.6	1	-	
2^nd ^quartile	24.5	1.62	1.17–2.24	
3^rd ^quartile	24.8	1.59	1.10–2.29	
4^th ^quartile	25.1	1.39	0.94–2.05	
**Social integration**				< 0.001
Isolated	12.0	1	-	
Helped	4.9	1.51	0.79–2.88	
Integrated	83.1	2.31	1.54–3.47	
**Lives in a couple relationship**				0.020
No	37.8	1	-	
Yes	62.2	1.35	1.05–1.73	
**Current health problem(s)**				0.007
No	34.9	1	-	
Yes	65.1	1.44	1.11–1.87	
**Relative with current health problem(s)**				0.002
No	48.3	1	-	
Yes	51.7	1.44	1.14–1.82	
**Perception of mental health**				0.024
Good	18.7	1	-	
Poor	81.3	1.41	1.05–1.91	
**Worries about health more than others**				0.040
No	75.9	1	-	
Yes	24.1	1.32	1.01–1.71	
**Difficulties understanding information from GP**				0.049
No	52.2	1	-	
Yes	47.8	1.27	1.01–1.60	
**Frequency of Internet use**				< 0.0001
Low	22.2	1	-	
High	77.8	2.96	2.15–4.07	
**Home connection**				0.014
No	21.4	1	-	
Yes	78.6	1.47	1.08–1.99	
**Years with access**				< 0.001
< 1	12.9	1	-	
1–3	28.9	1.37	0.94–2.02	
4–6	35.7	1.63	1.11–2.41	
> 7	22.5	2.45	1.60–3.76	

* Adjusted odds ratio

** Overall p_value based on the Type III Wald statistic

## Discussion

These analyses performed on a representative random sample of the population of the Paris metropolitan area confirm the main factors (age, level of income and education) discriminating Internet use in the general population on the one hand [2,3,30,31] and Internet use for seeking answers to health questions in the Internet user population on the other [7,32-34]. Our study shows simultaneously – in a single representative sample of the general population – an association with the three variables mentioned above for both outcomes. Researchers sometimes present ethnicity as a factor associated with health information seeking [35]. In France, it is illegal to ask people for their ethnicity, but it is allowed to ask for their nationality. Even if both do not explore the same dimensions, obviously, our data show that nationality is associated with Internet access but no longer with health information seeking among Internet users. In addition, new discriminating factors were observed. Specifically, our analyses show that social isolation is associated with a lower probability of Internet use. In the subsample of Internet users, women, people concerned about a health problem (their own or that of a close relative), those worried about their health or those who had difficulty understanding advice from physicians were more likely to have previously sought health information online than the others. The same is true for the individuals who were well integrated socially and who had Internet experience.

Even if the random sample from the Paris metropolitan area exhibits good representativeness, these results cannot be extended to the entire population of France because the Paris metropolitan area has specific characteristics not shared by the rest of the country. On average, its inhabitants are younger and have a higher level of education and a higher socio-occupational status [36]. Certain contextual factors themselves are different. Urban density, physician density [37] and the high-speed Internet connection rate [2] are notably higher there than in the other parts of France.

In addition, certain analyses performed on this sample of 3023 people may have suffered from a lack of statistical power because of the small sample size in some of the subgroups. However, the modelling performed on the 200 bootstrap samples generated from the initial sample shows that the results presented here are robust and satisfactorily adjusted to the data.

Our study confirms that the Internet penetration rate is higher in the Paris metropolitan area than it is nationally. Close to 60% of the respondents had a home connection, and 70% had previously personally gone online. In another national survey, the same rates were observed in Paris, while the estimates for France as a whole were respectively 43% and 54% during the same survey year [30]. These estimates are of the same order of magnitude as in all the other countries with high Internet penetration rates (65 to 75% in the United States, Japan and Sweden, for example [1]).

Furthermore, this study shows the existence, in the Paris metropolitan area, of a digital divide previously identified in France [2,30] and in other industrialised countries [4,31]. The probability of having Internet access decreases with age but increases with the level of education and income. The gender effect sometimes reported in Europe or the United States [38,39] was not observed in our study. These disparities are reinforced by the individuals' perception of their own socioeconomic situation. For a given level of education and income, the probability of having Internet access is lower in cases where there are perceived financial problems. Lastly, difficulty reading French also seems to be a barrier to Internet use. In general, people who do not have Internet access are those who have the least favourable social and economic characteristics: low income, no or few degrees, unemployed, foreign nationality and social isolation. Yet, these populations are also recognised as being more on the fringes of the health-care system and in less good health [40-42]. In our study, when adjusted for all of the socioeconomic characteristics, sick people also use the Internet less than others.

The analysis performed on the subsample of Internet users shows that nearly 49% of the individuals surveyed had previously searched online for information on a health-related topic during the previous three years. It is difficult to compare this estimate with those in other studies because the definition of the term "health" or the time period considered differs substantially from study to study. In late 2005, 28% of the Internet users in France had used the Internet to search "for information on health, a disease or diet and nutrition during the previous month" [30], while 58% of Norwegians [43] or 71% of European Internet users [7] had previously conducted online searches "for health purposes". In the United States, even though the total number of Internet users increased between 2004 and 2006, the proportion of "health seekers" remained stable (around 80% of American Internet users) [44].

With regard to socioeconomic status, certain determining factors of Internet access also discriminate health information seeking within the Internet user population itself. As has been shown in several international studies (often by descriptive or univariate analyses), the probability of having used the Internet to obtain health information decreases with age but seems to increase with the level of education and income [6]. On the other hand, the effect of the level of income seemed to distinguish between households with the poorest quartile of income and the others. While both the reported level of income and the subjective perception of financial difficulties, as well as occupational status, nationality and origin were factors discriminating Internet access, the second analysis revealed a significant association only between health information seeking and the actual level of household income. The data did not show a significant association with the subjective perception of one's socioeconomic status, occupational status or nationality.

As in other studies [7,30,33,34] (although some of them report discordant results [45]), our results show that women Internet users are the most active online health seekers. In general, women can be considered the ones who usually look after health matters in their families [46]. Although our data do not show a significant association with the number of children, a positive relationship is observed with living in a couple relationship or there being a sick individual in one's close circle. The gender effect appears perhaps to manifest as a different level of interest in health.

Some authors suggest that, in fact, the health information available online particularly benefits the already privileged in terms of health and health-care utilisation and/or the well-educated [10-12]. Indeed, a European study found that the probability of health information seeking grows with the number of visits to a physician [7]. In the French context of universal health insurance, our multivariate analysis did not show the four variables concerning health-care system utilisation or health insurance coverage to have a significant effect. On the other hand, our analyses did show strong associations between Internet use for seeking answers to health questions and health status, experiences and perceptions. As some studies tend to show with perceived health status [47,48] or long-term illness [7,8], the probability of previously having searched for health information online was greater in people who were sick and in those with a poor perception of their mental health status and/or who had a close relative with an illness. A positive association was found among individuals who feel that they worry more about their health than others, which confirms the notion in a previous American study which found that health seekers were "more likely to be health-oriented" [9]. As for health status (reported), the opposite effect was observed for our first outcome. While the sick were less likely to have Internet access, it was they who, among the Internet users, used this tool more often to obtain health information. The Internet therefore seems to be an important information-seeking tool for people dealing with an illness, when they have an opportunity to go online.

To understand and interpret our results more globally, we can refer to the concept of eHealth literacy [18,19], which combines the dimensions that underlie health literacy (functional, critical and interactive) [49] and online skills [50]. Although having reading difficulties appears to be a barrier to Internet use, we did not observe any effect associated with health information seeking in the Internet user population. The analysis of each of the outcomes shows postsecondary education to have a positive effect. Several studies do, in fact, show that the contents of health-related websites are written in a language geared to a high level of education [11].

All factors otherwise being equal (and especially for a given level of education), the probability of having used the Internet to seek answers to health questions is greater in individuals who find information provided by physicians difficult to understand. On the other hand, having difficulty applying health advice in daily life (adjusted only for gender, age, level of education and level of income) no longer appears to be significantly associated with online health information seeking in the final model. It may thus be asked to what extent the Internet is useful to people with poor health literacy.

Lastly, a double socioeconomic divide was previously reported in another study concerning Internet access and general Internet use (but not specifically health information seeking) [31]. Our data show that similar factors (age, level of education and level of income) are associated both with Internet access and the use of the Internet for health information searching. Furthermore, our study shows that Internet experience has a positive effect. Health information seeking appears to be more common when the frequency of use and the number of years of Internet use is higher. It seems that, with experience, the Internet assumes an increasingly important role in Internet users' lives in terms of how they obtain information and can even become an integral part of their daily lives. Searching on the Internet would thus become a habit or even an automatic reflex when searching for any information in general, just as when searching for health information in particular.

## Conclusion

In the context of France's universal health insurance program and of high physician density in the Paris metropolitan area, access to the Internet varies according to social and socioeconomic status and health status, and its use for health information seeking varies also with health beliefs, but not to health insurance coverage or health-care utilisation. Certain social and economic inequalities seem to impact cumulatively on Internet access and on its use to seek health information. The analyses performed on a representative sample from the Paris metropolitan area show that the most disadvantaged social groups and those the least integrated socially use the Internet less and, in the Internet user population itself, are again those that seek health information on the Internet the least.

We found that people who would need the Internet the most as a potential source of health information – to compensate for a lack of information or for remoteness from the health-care system (difficult economic circumstances, social isolation, health problems) – are also those who use it the least. Among Internet users, those furthest removed from health concerns (the non-sick or the less health-concerned) search for health information online less. It is therefore not obvious that the Internet is a special information tool for primary prevention. On the other hand, for patients and individuals who have difficulty understanding information provided by physicians, the Internet appears to be a useful complement for secondary prevention, especially for better understanding health problems or enhancing therapeutic compliance.

## Competing interests

The author(s) declare that they have no competing interests.

## Authors' contributions

ER participated in conceiving and designing the study and in acquiring the data, performed analyses, interpreted the results, and participated in drafting and revising the manuscript. IP participated in conceiving and designing the study and revised the manuscript. PC participated in conceiving and designing the study, in interpreting the results and in revising the manuscript. All three authors approved the final manuscript.

## Pre-publication history

The pre-publication history for this paper can be accessed here:


